# Biological Suppression of Populations of *Heterodera schachtii* Adapted to Different Host Genotypes of Sugar Beet

**DOI:** 10.3389/fpls.2020.00812

**Published:** 2020-06-19

**Authors:** Caroline Eberlein, Holger Heuer, Andreas Westphal

**Affiliations:** ^1^Kearney Agricultural Research and Extension Center, Department of Nematology, University of California, Riverside, Parlier, CA, United States; ^2^Institute for Epidemiology and Pathogen Diagnostics, Julius Kühn-Institut, Braunschweig, Germany

**Keywords:** augmentation, biocontrol, *Heterodera schachtii*, sugar beet cyst nematode, soil suppressiveness

## Abstract

Productivity of sugar beet and brassica vegetable crops is constrained by the nematode *Heterodera schachtii* worldwide. In sugar beet cropping areas of Central Europe and North America, *H. schachtii* is managed by crop rotation, and cultivation of resistant brassica cover crops. The recently released nematode-tolerant sugar beet cultivars suffer less damage than susceptible cultivars at high initial population densities of *H. schachtii*. Many tolerant cultivars allow for less nematode reproduction than susceptible cultivars. Monoculture of susceptible hosts can facilitate the evolution of suppressive soil. Objectives of this study were to determine if susceptible hosts are required for this process, and if monoculture with sugar beet genotypes of different host status (susceptible, resistant, tolerant) impact this capacity. Additionally, we tested if amending soil with the cyst nematode pathogens *Pasteuria nishizawae* or *Hyalorbilia* sp. strain DoUCR50 favored the establishment of soil suppressiveness. In 4-year microplot studies with *H. schachtii* Schach0 or Schach1, one susceptible, one Schach0-resistant, and one tolerant sugar beet genotype were monocultured. In 2010, plots were amended with *P. nishizawae* or DoUCR50, the last being introduced into non-treated soil for Schach0, and into previously biocide-treated soil for Schach1. In 2011, respective Schach0 plots received a second amendment with DoUCR50. Nematode population densities and growth and yield parameters were determined annually. Effects of *P. nishizawae* and DoUCR50 on populations of *H. schachtii* were limited and not consistent. Starting in the second year of the monoculture, eggs of both *H. schachtii* pathotypes became diseased. Up to 90% of the total eggs were encumbered by the third cropping cycle, under the susceptible, resistant, and tolerant cultivar. In all years, the tolerant genotype produced the highest and most stable white sugar yields while yields of the other cultivars slowly improved during the monoculture. Results of this study suggested the presence of egg-infecting factors in this sugar beet monoculture that dramatically increased the proportions of diseased eggs. The tolerant cultivar allowed establishment of soil suppressiveness without the initial yield decline observed when susceptible sugar beet genotypes are grown in monoculture.

## Introduction

The soil environment is the place of tri-trophic interactions of the plant, its parasites/pathogens, and potential beneficial organisms/hyperparasites that interfere with activities of the second trophic level. In many cases, these interactions can result in suppression of the detrimental organisms allowing flourishing growth of host plants despite the presence of the parasites. In extreme cases of degraded soils, the balance of parasites and their antagonists is offset, and severe activities of detrimental organisms with resulting disease expression occur (Sikora, 1992; Stirling, 2014). Some suppression of parasitic activity by biological balancing is expected in most soils (Stirling, 1991; Mazzola, 2004; Janvier et al., 2007). The discovery of soils where this antagonistic potential or soil suppressiveness is noticeably high has fueled interest in biological control mechanisms in the soil environment. Several studies have reported such soils, and methods of their detection and description have long been proposed (Weller et al., 2002; Westphal, 2005; Borneman and Becker, 2007).

The currently accepted hypothesis is that monoculture of a susceptible host is mandatory for generating such pathogen-suppressive soils. This train of thought was based on results from early studies on take-all decline soils that established under wheat monoculture (Cook, 1981), and also from investigations of cereal cyst nematode suppressive soils in the United Kingdom (Gair et al., 1969). The recommendation of using a susceptible host seemed supported by the lack of increase of population densities of the antagonists under a resistant cereal crop (Kerry and Andersson, 1983). Monoculture of a susceptible host has repeatedly been viable to generate suppressive soil, presumably when beneficial organisms for induction of this phenomenon were present at the beginning of such strategy (Gair et al., 1969; Baker and Cook, 1974; Westphal and Becker, 1999; Chen, 2007). It appeared that at least one of the target pathogens needed to be present at onset of such trials to result in suppressive soil (Westphal and Xing, 2011).

The practical value of generating suppressive soil by monoculture of susceptible hosts was quickly negated because of the expected yield losses in such strategy that rendered this approach non-viable for commercial producers (Kerry, 1990). The role of resistant cultivars as impacting this balance within this tri-trophic network in suppressive soil has been studied (Westphal and Becker, 2001). In that study, resistant cultivars of *B. vulgaris* or *Raphanus sativus* were able to preserve suppressiveness against the sugar beet cyst nematode while a double crop of the non-host *Triticum aestivum* reduced soil suppressiveness against *Heterodera schachtii* (Westphal and Becker, 2001). It was speculated that some activity of the nematode under the host crop was necessary for maintenance of this beneficial soil function. The hypothesis that tolerant cultivars may aid in the monoculture approach of host plants was formed many years ago but not tested so far.

In sugar beet production, recently cultivars tolerant to *H. schachtii* damage have been released, and quickly have become the preferred sugar beet genotypes in high-production areas. These cultivars can withstand higher population densities of *H. schachtii* than standard susceptible (and sensitive) cultivars. Under high population densities but also under very low population densities, tolerant sugar beet cultivars are able to have high yields, and almost identical to non-tolerant high yielding cultivars under non-infested conditions (Heinrichs, 2013; Kaemmerer et al., 2014). In contrast, resistant cultivars have a lower yield potential than tolerant or susceptible cultivars, especially in the absence or under low population densities of the respective nematode, where they can yield 15% less compared to susceptible cultivars (Schlinker, 2010, 2012; Bürcky, 2013). Tolerant cultivars do permit some reproduction higher than resistant cultivars, but less than susceptible ones (Westphal, 2013; Kaemmerer et al., 2014). Such genotypes potentially offer opportunity to overcome the yield decline in the “establishing phase” of soil suppressiveness.

It was our hypothesis that these tolerant genotypes would not suffer as severe yield losses in the initiating time of the suppressiveness as susceptible lines while permitting significant increases of suppressive principals in the soil. This was challenging the current concept of mandatory susceptible crop monoculture. As representative model organisms for an obligate parasite, *Pasteuria nishizawae* was included in these trials. *Hyalorbilia* aff. *multiguttulata* DoUCR50 (DoUCR50, NCBI GenBank accession number JQ638668), closely related to *Hyalorbilia oviparasitica* (formerly: *Dactylella oviparasitica*) represented highly effective but non-obligate fungal antagonists (Olatinwo et al., 2006b; Baral et al., 2018). The specific objectives of this study with *H. schachtii* pathotypes Schach0 (wild type with no virulence on any resistance sources) and Schach1 (virulent on sugar beet genotypes including those that carry the Hs1^pro–1^ gene for resistance to *H. schachtii*; Müller, 1998) were to determine: (A) if monoculture of sugar beet genotypes of different host suitability to *H. schachtii* (susceptible, resistant, tolerant) had similar effects on the development of soil suppressiveness and (B) if the obligate bacterial hyperparasite *P. nishizawae* or the facultative fungus DoUCR50 reduce nematode population densities and protect yield of susceptible, resistant, and tolerant cultivars.

## Materials and Methods

A multi-year study was conducted in outside microplots of 1 m^2^ surface area containing sandy soil (88.2% sand, 7.4% silt, 4.4% clay, 2.4% O.M., pH 6.9) that had been originally infested with *H. schachtii* pathotype Schach0 (16 microplots) or Schach1 (16 microplots) at Münster, Germany. Plots had been used for crop rotation and nematode management research on *H. schachtii* for multiple years before initiating the current project. For each pathotype, a different experiment was conducted. Each experiment was arranged as a split-plot design with the entire microplots serving as mainplots, further divided into three subplots with a total of four replicates. In April 2010, main plots infested with Schach0 received the following treatments: (i) untreated control (ii) *P. nishizawae* amendment, (iii) DoUCR50 amendment, and (iv) an experimental nematicidal seed treatment (only applied in 2010). Mainplots infested with Schach1 received the following treatments: (i) untreated control, (ii) *P. nishizawae* amendment, (iii) Dazomet + DoUCR50 amendment, and (iv) Dazomet—DoUCR50 (no amendment). On 7 April 2010, dazomet at 500 kg/ha (tetrahydro-3,5-dimethyl-1,3,5-thiadiazine-2-thione; Basamid, BASF, Germany) was applied to the soil surface and incorporated following general label instructions before covering the plots with 0.04 mm thick black PE tarp (Polydress). This was done to perturb soil microbial communities. These differences in treatment among the two experiments were done because the Schach1 population was expected to be more difficult to suppress on two of the three sugar beet cultivars being used. On 30 April 2010, microbial treatments were applied. Within each mainplot, the three parallel subplots (33 × 100 cm) for one three-plant row each were established in split-plot design. For microbial amendments, a soil core of 19-cm diameter and 10-cm depth was removed from each of the three planting sites per subplot, and the total 8.5 L were transferred into a 20-L bucket. The soil was mixed and amended with either 150 mL DoUCR50 suspension (the equivalent of three 3-week-old 9-cm potato dextrose agar culture plates incubated in the dark at room temperature; approximately 3.5 × 10^7^ CFUs per subplot), or 3.04 g *P. nishizawae* spores (4.25 × 10^9^). DoUCR50 suspension was produced according to the method by Olatinwo et al. (2006b). *P. nishizawae* inoculum was commercially provided (Pasteuria Bioscience, Inc., Alachua, FL, United States). The respective soil mixes were then evenly distributed and replaced into the three planting sites per subplot. Two days after the amendments, 0.3 m × 1.0 m subplots were randomly assigned to *B. vulgaris* L. ‘Beretta’ (susceptible to *H. schachtii*), ‘Sanetta’ (resistant to Schach0), or ‘Pauletta’ (tolerant to *H. schachtii*). At each of the three planting sites per subplot, six seeds of the respective cultivar were hand seeded. For control plots without microbial amendments, the same soil mixing procedures were followed. In 2011, in Schach0-infested plots, plots amended with DoUCR50 received a second amendment (3.5 × 10^7^ CFUs per subplot), whereas plots that were planted to treated sugar beet seeds in 2010 were amended with DoUCR50 (3.5 × 10^7^ CFUs per subplot). Both experiments, Schach0 and Schach1, were cropped in monoculture of sugar beet with the same sugar beet genotypes in each subplot until harvest 2013. During the vegetation period, the patterns of temperature (14.1–14.9°C) and precipitation (290–480 L/m^2^) varied between years (Supplementary Figure S1). Plots were carefully monitored, and at beginning of water stress, supplement irrigation was administered as needed to sustain unimpeded plant growth.

In both experiments, approximately three weeks after sowing at the cotyledon stage with initial true leaves (Biologische Bundesanstalt, Bundessortenamt und Chemische Industrie, BBCH 10; Meier et al., 1993), one seedling per planting site was removed, so that from each replication, three seedlings per cultivar in each treatment were removed for staining. One seedling was maintained per planting site in the plots, the rest was discarded. After staining the roots with acid fuchsin (Byrd et al., 1983), root penetration by second-stage juveniles (J2) was determined. The root lengths of the seedlings were measured with a ruler and penetration was reported as J2 per centimeter of root. Every year prior to sowing and during the growing season, plots were fertilized with varying combinations of NPK (12% N, 5.2% P, 6.6% K), ammonium nitrate (27% N) and thomaskali (3.5% P, 12.5% K) fertilizers to deliver N rates ranging from 40 to 120 kg ha^–1^, P rates of 120 kg ha^–1^, and K rates of 170 kg ha^–1^. Plants were maintained following standard regional cultivation recommendations including watering as needed, and fungicide and insecticide applications. To control insect pests, 0.05% Fenpropathrin (Rody, Sumitomo Chemical Co. Ltd., Japan) was sprayed, and Imidacloprid (Confidor 70 WG, Bayer CropScience, Germany) was applied as soil drench. These insecticides are not known to reduce fungal activities. To control fungal pathogens, 1 L/ha Difenoconazol/Fenpropidin (Spyrale, Syngenta Crop Protection AG, Switzerland) was sprayed, care being taken to avoid run-off into the soil. Plant growth was monitored and the perpendicular diameter of the plant canopy was measured 6–9 weeks after sowing. At harvest, fresh weights of washed sugar beet taproots were determined. Sugar content was determined by standard procedures (Buchholz et al., 1995) and reported as white sugar yield (WSY) per plant.

To determine initial population densities of *H. schachtii* prior to sowing, soil samples of 12 2-cm diameter cores were collected per subplot from the upper 30 cm with a soil corer. No such samples were processed for the experiments in 2013. To determine final nematode population densities at harvest, four 2-cm diameter soil cores were taken from the root zone of each of the three plant sites of one subplot. Twelve soil cores per subplot were separated into 0–30- and 30–60-cm depth samples. Subsamples of 400 g of fresh soil were used for extracting cysts by density centrifugation with MgSO_4_ (Müller, 1980). Cysts were counted under a binocular before being crushed in a custom-made tissue grinder to release the eggs and juveniles. These nematode stages were suspended in water and counted in duplicate under an inverted transmitted light microscope (63× magnification), using 2 × 1 mL aliquot portions. During counting, eggs and juveniles were classified into healthy (normally developed, intact J2 inside) or diseased (abnormal development or obviously colonized by microbes).

### Statistical Analysis

Analysis of variance was carried out using a four-factor split-plot model including experimental year in SAS (version 9.4, SAS Institute, Cary, NC, United States). Analyses were performed for each experiment separately. In the spring, nematode numbers were only from 0 to 30 cm depth (only 2010–2012 data), at harvest, numbers of the depth 0–30 and 30–60 cm were included in the model as a strip factor within the split-plot model. Count data was log_10_-transformed [log_10_(x + 1)]. For each year, pooling of error terms was done where possible to simplify models in Proc GLM. Regression analysis was conducted with PROC REG and slope comparison was done in PROC GLM (Rasch and Verdooren, 2004). To compare nematode populations, growth, and yield parameters between years, data were analyzed as repeated measurements using the GLIMMIX procedure of SAS (version 9.4). Specific comparisons were tested and their *P*-values adjusted with the Edwards and Berry’s simulation method. Statistical significance was set at *P* ≤ 0.05. Results obtained from Proc GLIMMIX are presented as back-transformed lsmeans ± lsmse.

## Results

The main factors year and cultivar significantly impacted all parameters measured (Tables 1, 2). Egg population densities and yields changed over the years and the factor year interacted with several other factors as described in more detail below. In both experiments, nematode population densities were stratified by depth, which was also impacted by year (Tables 1, 2). In Schach1, population of healthy eggs at the 0–30 cm soil depth significantly decreased over time (from 8365 eggs/100 g of soil to 266 eggs/100 g of soil), with a decline to less than half in the second year of the experiment. In the last two years, number of healthy eggs were close to the detection level (62–266 eggs/100 g of soil; data not shown). At the 30–60 cm soil depth, population of healthy eggs decreased following the same pattern of eggs at the 0–30 cm depth, but with initial numbers much lower than at the 0–30 cm soil layer (3074 eggs/100 g of soil; data not shown). In both nematode pathotypes, populations of total eggs at both soil depths decreased in the third year of the experiment to then reach final numbers by the end of the experiment that were still lower than the initial numbers in 2010, with the exception of eggs at the 30–60 cm depth in Schach0 with final numbers similar to those in 2010 (data not shown). Because the depth factor only interacted with the year factor, foremost the average of nematode numbers in 0–30 and 30–60 cm depth were used for illustrating data in more detail below.

**TABLE 1:** Summary of ANOVA *P*-values of four-factor effects on early plant growth, early root invasion by nematodes, initial and final egg population densities and health, and sugar yield in an experiment with sugar beet and *Heterodera schachtii* Schach0 at Münster, Germany from 2010 to 2013^a^.

			**Egg population density**						
	**Early season**		**Planting**			**Harvest**			**White sugar yield**
**Factor**	**Diameter**	**J2/root**	**Healthy**	**Diseased**	**Total**	**Healthy**	**Diseased**	**Total**	
Treatment (TRT)	0.0986	0.0855	0.2211	0.2071	0.1705	0.1745	0.2471	0.1564	0.0337
Cultivar (CV)	<0.0001	<0.0001	0.0010	<0.0001	<0.0001	<0.0001	<0.0001	<0.0001	<0.0001
TRT × CV	0.4200	0.1390	0.2473	0.1865	0.1740	0.0425	0.0200	0.0411	0.0010
Depth (D)	–	–	–	–	–	0.0031	0.0017	0.0015	–
Year (Y)	<0.0001	<0.0001	<0.0001	<0.0001	<0.0001	<0.0001	<0.0001	<0.0001	<0.0001
TRT × Y	0.0990	0.0212	0.4019	0.0002	0.3459	0.1031	0.3284	0.3526	0.9597
CV × Y	0.1443	0.5123	0.2601	0.3274	0.9112	0.4503	0.0110	0.0056	<0.0001
TRT × CV × Y	0.8763	0.4627	0.8806	0.6526	0.8466	0.6873	0.8038	0.6409	0.6440
D × Y	–	–	–	–	–	0.1309	<0.0001	<0.0001	–

*^a^Interactive factors that were non-significant for all parameters in both experiments were not shown.*

**TABLE 2:** Summary of ANOVA *P*-values of four-factor effects on early plant growth, early root invasion by nematodes, initial and final egg population densities and health, and sugar yield in an experiment with sugar beet and *Heterodera schachtii* Schach1 at Münster, Germany, from 2010 to 2013^a^.

			**Egg population density**						
	**Early season**		**Planting**			**Harvest**			**White sugar yield**
**Factor**	**Diameter**	**J2/root**	**Healthy**	**Diseased**	**Total**	**Healthy**	**Diseased**	**Total**	
Treatment (TRT)	0.5088	0.7216	0.0029	0.0001	<0.0001	0.0041	0.0029	0.0006	0.6305
Cultivar (CV)	<0.0001	<0.0001	0.3166	<0.0001	<0.0001	0.0008	<0.0001	<0.0001	<0.0001
TRT × CV	0.5098	0.2886	0.5375	0.2658	0.0182	0.2966	0.0157	0.0032	0.5657
Depth (D)	–	–	–	–	–	0.0011	<0.0001	<0.0001	–
Year (Y)	<0.0001	<0.0001	<0.0001	<0.0001	<0.0001	<0.0001	<0.0001	<0.0001	0.0019
TRT × Y	<0.0001	<0.0001	<0.0001	0.0001	<0.0001	0.0554	0.0902	<0.0001	0.2179
CV × Y	0.6028	0.4845	0.1448	0.0869	0.0086	0.3932	0.4490	0.0207	0.0003
TRT × CV × Y	0.0976	0.0426	0.6974	0.6029	0.0835	0.2279	0.4540	0.0001	0.0037
D × Y	–	–	–	–	–	<0.0001	0.0044	<0.0001	–

*^*a*^Interactive factors that were non-significant for all parameters in both experiments were not shown.*

### Population Densities of *Heterodera schachtii* Under the Different Cultivars

In both experiments, differences in the final population densities at harvest were observed among susceptible (Beretta), resistant (Sanetta), and tolerant (Pauletta) cultivars according to their known host status (Tables 1, 2).

**FIGURE 1:**
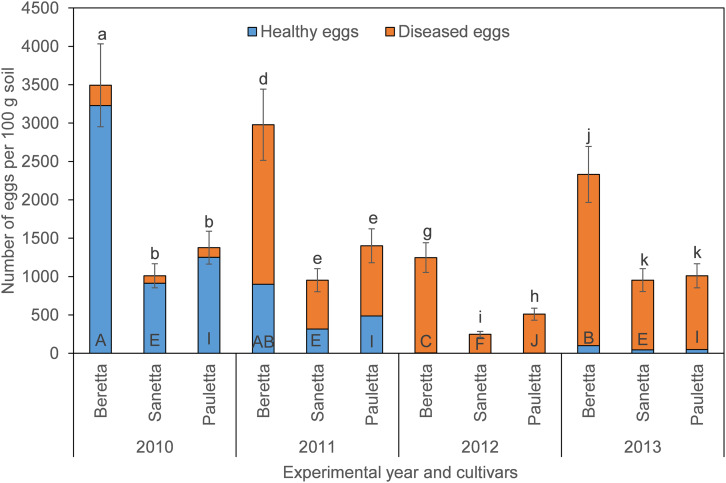
Final population densities of eggs of *Heterodera schachtii* Schach0 in Münster from 2010 to 2013 under sugar beet cropping (Beretta, Sanetta, Pauletta), and averaged across treatment and soil depth. Data are presented as back-transformed lsmeans ± lsmse. Bars within each year indexed with the same letter were not significantly different at *P* = 0.05; a–c: 2010; d–f: 2011; g–i: 2012; j–l: 2013. Bars within each cultivar over year indexed with the same letter were not significantly different at *P* = 0.05; A–D: Beretta; E–H: Sanetta; I–L: Pauletta.

In Schach0, final population densities of total eggs decreased over time under Beretta, but remained similar under Sanetta and Pauletta (Figure 1). Overall, numbers of total eggs were higher under Beretta than Sanetta and Pauletta, the last two showing similar numbers, except in 2012, where numbers were higher under Pauletta than Sanetta (Figure 1). The proportion of healthy eggs decreased per year under the three cultivars after the first year (Table 1 and Figure 1). In Beretta, this reduction in healthy eggs was more pronounced from the first to the second year (Figure 1). In 2012 and 2013, healthy eggs under all cultivars were close to detection levels (Figure 1). Separate graphs for number of healthy, diseased, and total eggs are provided as Supplementary Figure S2.

**FIGURE 2:**
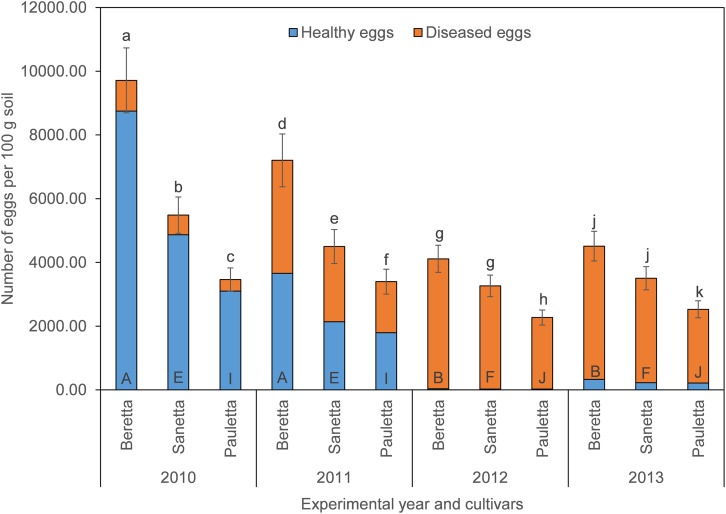
Final population densities of eggs of *Heterodera schachtii* Schach1 in Münster, from 2010 to 2013 under sugar beet cropping (Beretta, Sanetta, Pauletta), and averaged across treatment and soil depth. Data are presented as backtransformed lsmeans ± lsmse. Bars within each year indexed with the same letter were not significantly different at *P* = 0.05; a–c: 2010; d–f: 2011; g–i: 2012; j–l: 2013. Bars within each cultivar over year indexed with the same letter were not significantly different at *P* = 0.05; A–D: Beretta; E–H: Sanetta; I–L: Pauletta.

In Schach1, final populations of total eggs decreased over time similarly under Beretta, Sanetta, and Pauletta (Figure 2). In the first two years, numbers of total eggs were higher under Beretta than under Sanetta and Pauletta, the latter with lower numbers than Sanetta (Figure 2). In the last two years, numbers of total eggs among Beretta and Sanetta were similar and higher than under Pauletta (Figure 2). Under all cultivars, the proportion of healthy to diseased eggs declined over time (Table 2 and Figure 2). The reduction in healthy eggs was most pronounced under the susceptible cultivar Beretta (Figure 2). In 2012 and 2013, numbers of healthy eggs were close to the detection limit (Figure 2). Separate graphs for number of healthy, diseased, and total eggs are provided as Supplementary Figure S3.

### Population Densities of *Heterodera schachtii* Under the Different Soil Treatments

**FIGURE 3:**
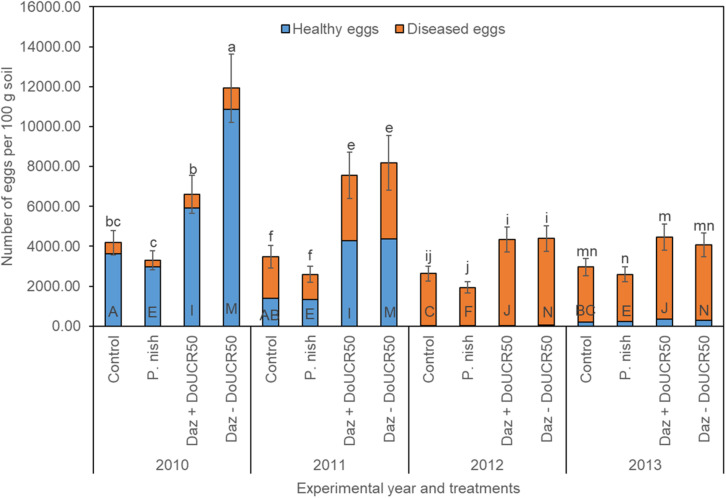
Final population densities of eggs of *Heterodera schachtii* Schach1 in Münster, from 2010 to 2013 under four different treatments averaged across cultivar and soil depth. Data are presented as backtransformed lsmeans ± lsmse. Bars within each year indexed with the same letter were not significantly different at *P* = 0.05; a–d: 2010; e–h: 2011; i–l: 2012; m–p: 2013. Bars within each treatment over year indexed with the same letter were not significantly different at *P* = 0.05; A–D: untreated control (Control); E–H: *P. nishizawae* (*P. nish*); I–L: Dazomet + *Hyalorbilia* sp. strain DoUCR50 (Daz + DoUCR50); M–P: Dazomet - *Hyalorbilia* sp. strain DoUCR50 (Daz – DoUCR50).

When averaging across cultivars and examining treatment effects for Schach1, final populations of total eggs decreased over time under the untreated control, dazomet + DoUCR50, and dazomet – DoUCR50, but remained similar under the treatment with *P. nishizawae* (Figure 3). Whereas in the first year, numbers of total eggs were higher after treatment with dazomet – DoUCR50, compared to dazomet + DoUCR50, from the second until the last year, numbers were similar among these two treatments (Figure 3). When considering the interaction between cultivar and treatment over time, numbers of total eggs in the first year were higher after treatment with dazomet – DoUCR50 than after inoculation with DoUCR50 but only under Sanetta (data not shown). Under Beretta, total eggs were numerically higher after treatment with dazomet – DoUCR50 than after dazomet + DoUCR50 (data not shown). Numbers of total eggs after amendment with *P. nishizawae* were similar to the untreated control in all years (Figure 3). Proportion of healthy eggs diminished over time, being close to the detection level during the last two years (Figure 3). Proportions of diseased eggs increased over time (Figure 3). Separate graphs for number of healthy, diseased, and total eggs are provided as Supplementary Figure S4.

### White Sugar Yield of Sugar Beet in Monoculture of Three Different Cultivars

The cultivars had different yield potential in the nematode-infested soil. Average yields increased over years, and did so differently for the different cultivars. Whereas WSY was stable throughout the years for Pauletta, different dynamics with a yearly increase of WSY for Beretta and Sanetta were determined. Treatment effects on yield were somewhat limited.

**FIGURE 4:**
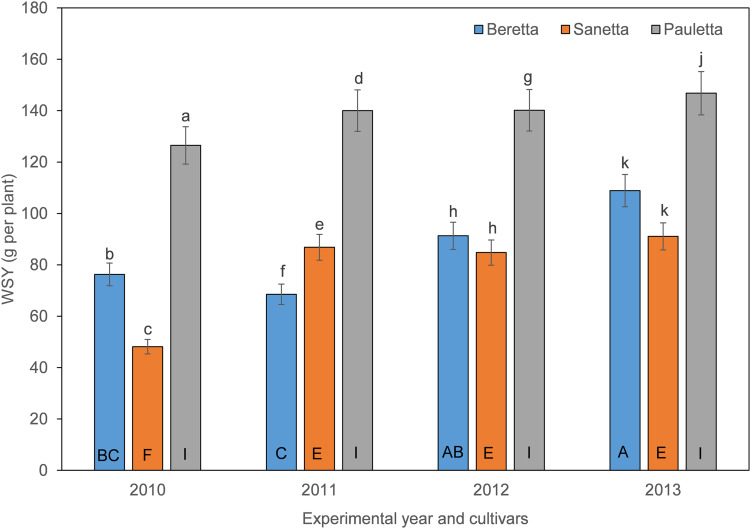
White sugar yield (WSY) of three different cultivars of sugar beet (Beretta, Sanetta, Pauletta) grown in microplots infested with *Heterodera schachtii* Schach0 in Münster from 2010 to 2013. Data are averaged across treatment and are presented as back-transformed lsmeans ± lsmse. Bars within each year indexed with the same letter were not significantly different at *P* = 0.05; a–c: 2010; d–f: 2011; g–i: 2012; j–l: 2013. Bars within each cultivar over year indexed with the same letter were not significantly different at *P* = 0.05; A–D: Beretta; E–H: Sanetta; I–L: Pauletta.

**FIGURE 5:**
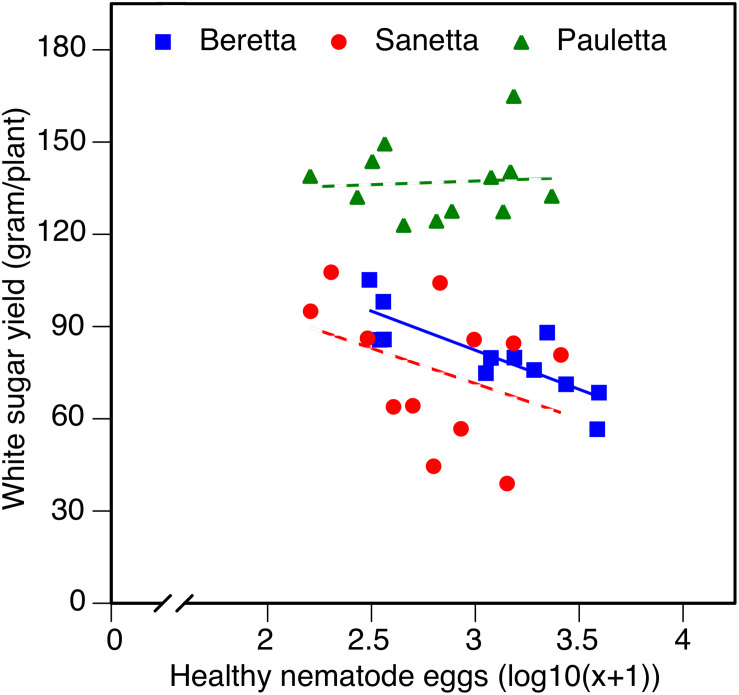
White sugar yield of sugar beet in relation to healthy nematode eggs at planting in *Heterodera schachtii* Schach 0-infested soil in 2010–2012. Data are averaged across treatment. Solid line: Beretta *f*(*x*) = –25.4395 *x* + 158.6060, *R*^2^ = 0.64; *P* < 0.01; hatched line: trendlines for Sanetta and Pauletta.

In Schach0, WSY was stable and higher in Pauletta than in the other two cultivars and did not change over time (Figure 4). WSY of Beretta declined slightly from 2010 to 2011 to then increase steadily until the last year of the experiment (Figure 4). In 2010, the lowest WSY was found in Sanetta. To the second year, Sanetta WSY increased and remained constant at that level (Figure 4). In 2011, Sanetta WSY was higher than in Beretta. In 2012 and 2013, WSY was similar in Beretta and Sanetta (Figure 4). Only for Beretta, a significant negative linear regression for WSY and healthy nematode eggs at planting was ascertained [Beretta *f*(*x*) = –25.4395 *x* + 158.6060, *R*^2^ = 0.64; *P* < 0.01; Figure 5). There was only a non-significant trend line with a negative slop for Sanetta and Pauletta’s non-significant trendline (Figure 5). There were no significant differences in WSY among the soil treatments (Table 1).

**FIGURE 6:**
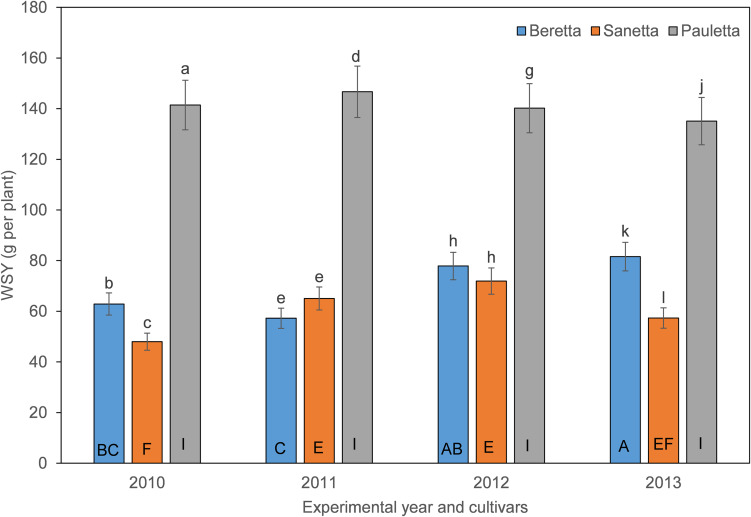
White sugar yield (WSY) of three different cultivars of sugar beet (Beretta, Sanetta, Pauletta) in microplots infested with *Heterodera schachtii* Schach1 in Münster from 2010 to 2013. Data are averaged across treatment and are presented as backtransformed lsmeans ± lsmse. Bars within each year indexed with the same letter were not significantly different at *P* = 0.05; a–c: 2010; d–f: 2011; g–i: 2012; j–l: 2013. Bars within each cultivar over year indexed with the same letter were not significantly different at *P* = 0.05; A–D: Beretta; E–H: Sanetta; I–L: Pauletta.

**FIGURE 7:**
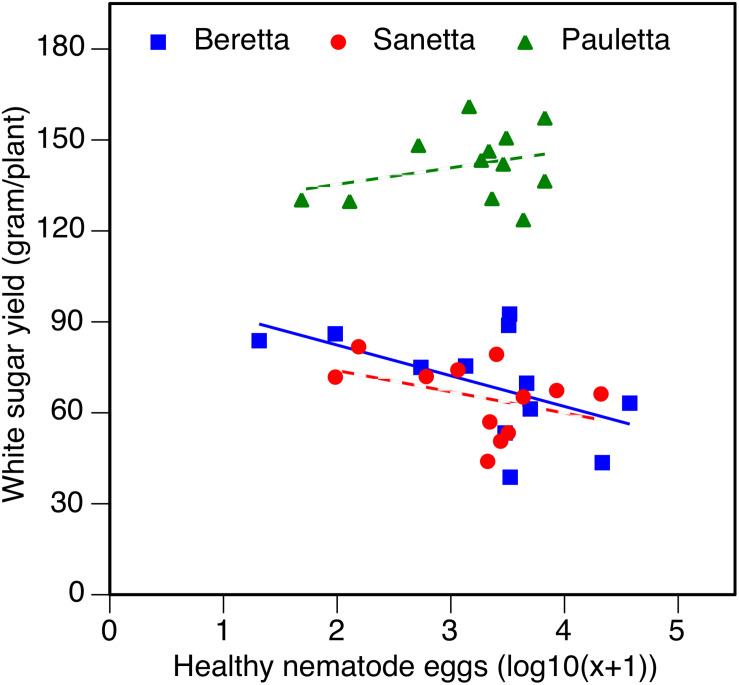
White sugar yield of sugar beet in relation to healthy nematode eggs at planting in *Heterodera schachtii* Schach1-infested soil at Münster in 2010–2012. Data are averaged across treatment. Beretta *f*(*x*) = –10.1271 *x* + 102.5928, *R*^2^ = 0.20; *P* = 0.0808.

In Schach1, the WSY in Pauletta remained constantly high over time above both other cultivars (Figure 6). WSY of Beretta and Sanetta were on a similar level in 2011 and 2012 but increased in Beretta over time while staying on a similar level in Sanetta throughout the monitoring time (Figure 6). Only for Beretta, a negative linear regression trend for WSY and healthy nematode eggs at planting was observed [Beretta *f*(*x*) = –10.1271 *x* + 102.5928, *R*^2^ = 0.20; *P* = 0.0808; Figure 7). There was only a non-significant trend line with a negative slop for Sanetta, and a non-significant level to slightly positive trendline for Pauletta (Figure 7). In this experiment, in the first year after Dazomet treatment before inoculation with DoUCR50, higher WSY compared to the non-treated control was found in Beretta, but numbers were similar to those under the Dazomet treatment only (data not shown). Yields did increase in the non-treated control in Beretta after 2010, and then were on a similar level with the other treatments for the remainder of the experiment. After amendment with *P. nishizawae*, WSY in the susceptible cultivar Beretta increased by the last year (data not shown).

### Sugar Beet Root Penetration and Impact on Early Canopy Diameter

Overall, the three cultivars followed a linear regression with a positive slope for the relationship of root penetration in relation to at planting-egg population densities in the soil. In the description of the relationship between juvenile root penetration and canopy diameter, a negative relationship was found.

**FIGURE 8:**
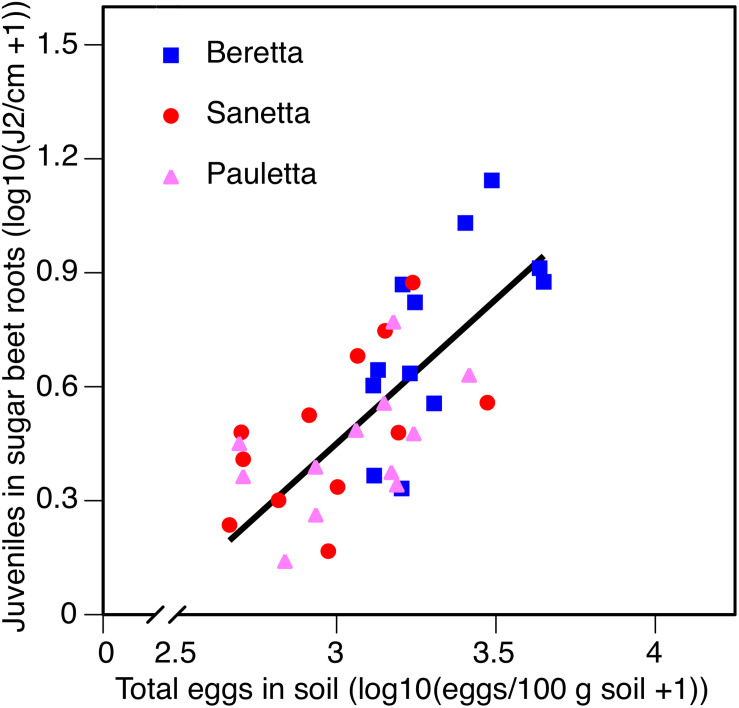
Number of juveniles (J2) in sugar beet roots in three sugar beet cultivars after four soil treatments in relation to total numbers of eggs of *Heterodera schachtii* at planting in Schach0-infested soil at Münster, Germany in 2010–2012.

In Schach0, averaged across treatments and years (2010–2012), the increase of root penetration with increasing numbers of eggs of *H. schachtii* in soil at planting was significant at *P* = 0.05 in Beretta but was not statistically different from the slopes in Sanetta and Pauletta that were only significant at *P* = 0.10 [Beretta *f*(*x*) = 0.8723 *x* – 2.1558; *R*^2^ = 0.38; *P* = 0.0189; Sanetta *f*(*x*) = 0.4806 *x* – 0.9552; *R*^2^ = 0.25; *P* = 0.0563; Pauletta *f*(*x*) = 0.4057 *x* – 0.7972; *R*^2^ = 0.22; *P* = 0.0689]. This interaction followed the linear regression *f*(*x*) = 0.7590 *x* – 1.8267; *R*^2^ = 0.50; *P* < 0.01 (Figure 8). Juvenile root penetration had only limited effects on the early canopy diameter (log-transformed) with just slight non-significant trends of decreasing diameters with increasing nematode population densities of the roots (data not shown). In all years, root penetration was numerically increased in Beretta than Sanetta and Pauletta (Supplementary Table S1), although this increase was not significantly different when considering the interaction between year and cultivar (Table 1). Overall, canopy diameter was numerically larger in Pauletta than Beretta and Sanetta (Supplementary Table S1) but not significant (Table 1).

**FIGURE 9:**
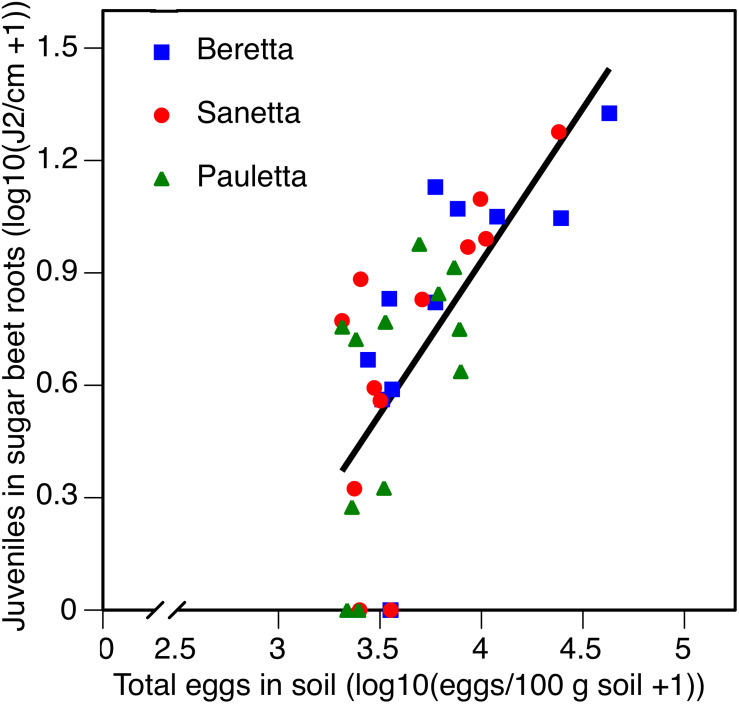
Number of juveniles (J2) in sugar beet roots in three sugar beet cultivars after four soil treatments in relation to total numbers of eggs of *Heterodera scchachtii* at planting in Schach1-infested soil at Münster, Germany in 2010–2012.

In Schach1, averaged across treatments and years 2010–2012, root penetration followed the same linear regression for all three cultivars *f*(*x*) = 0.8152 *x* – 2.3290; *R*^2^ = 0.45; *P* < 0.01 (Figure 9). Juvenile root penetration had a strong negative impact on canopy diameter (log-transformed). The mutual linear regression was described by *f*(*x*) = –0.3630 *x* + 1.8503; *R*^2^ = 0.61; *P* < 0.01 (data not shown). In all years, root penetration was more numerous in Beretta than Pauletta (Supplementary Table S1), although these differences were not significantly different at the year and cultivar interaction (Table 2). In every year, canopy diameter was numerically larger in Pauletta than Beretta and Sanetta (Supplementary Table S1), but not significantly (Table 2).

## Discussion

In this study, the monoculture of a *H. schachtii-*susceptible, resistant, and tolerant sugar beet cultivar led to severely declining health status of eggs of the nematode independent of the cultivar genotype. As expected, the tolerant cultivar produced the highest yields in plots of both pathotypes. The tolerant cultivar Pauletta maintained high yields throughout the monoculture while egg population health deteriorated. These data support the hypothesis that a tolerant cultivar is useful to protect from yield losses when initiating a host plant monoculture for developing soil suppressiveness. The hypothesis that some nematode activity was important for maintenance of suppressiveness (Westphal and Becker, 2001) and the need for the pathogen to be present (Westphal and Xing, 2011) seemed supported by these findings. The nematode health decline in all plots allowed for some increasing yields in the susceptible and resistant cultivars further illustrating the development of a suppressive soil.

Decline of *H. schachtii* population densities under a sugar beet monoculture was observed by Thielemann and Steudel (1973) in a field at Elsdorf, Germany, and by Heijbroek (1983) in the Netherlands. In the United Kingdom, Crump and Kerry (1987) found that there was little population increase of *H. schachtii* in the untreated soil during a three year-field trial. In our study, after four years of monitoring, the decline of Schach0 egg final populations under the susceptible cultivar (20%) was similar to the decline observed by Thielemann and Steudel (10%) and by Heijbroek (30%). In Schach1 however, eggs final populations under the susceptible cultivar decreased by 70% after four years. On the other hand, diseased eggs presenting fungal hyphae or physiologically disordered content that represented around 10% in 2010, increased by 2013 to >90% in Schach0 and Schach1. This pronounced increase in diseased eggs in both pathotypes suggests the presence of controlling agents in this sugar beet monoculture soil. High numbers of diseased eggs were also observed by Bursnall and Tribe (1974) in *H. schachtii*, by Morgan-Jones et al. (1981) in *H. glycines*, and by Dackman (1990), Eberlein (2017) and Eberlein et al. (2016) in potato cyst nematodes.

Westphal and Becker (2001) found that soil suppressiveness against *H. schachtii* was reduced after a double crop of the non-host *T. aestivum* but preserved after cropping of resistant cultivars of sugar beet or oil radish. Cotton, watermelon, and melon resistant cultivars to their respective Fusarium wilt, some of them grown in monoculture, have been shown to induce soil suppressiveness against the causal agent of this disease (Katan et al., 1983; Hopkins et al., 1987; Sneh et al., 1987). Hopkins et al. (1987) also found that the suppressiveness that developed after monoculture of a resistant watermelon cultivar was effective on all cultivars. It seems that resistant cultivars not only have the ability *per se* to reduce the population densities of a pathogen, but also indirectly by enhancing microbial antagonists that are able to survive under reduced populations of the pathogen, thus inducing the development of soil suppressiveness.

To enhance the potential for establishment success, plots of Schach1 were pretreated with dazomet to remove biological buffering before amending with DoUCR50. Such treatment had previously been useful to disturb microbial populations without eliminating cyst nematodes from field plots (Xing and Westphal, 2009). The lack of soil receptivity had been expected when introducing non-native organisms to soil, especially in such a different agro-ecological environment considering its’ presumed niche in California. Parasitism, competition for nutrients, and microbiostasis can negatively impact the establishment of nematode-parasitic fungi (Cook and Baker, 1983; De Boer et al., 2003; Monfort et al., 2006). In 2010, the Dazomet treatment resulted in a reduction of final populations of total eggs under the resistant cultivar, compared to the corresponding control (dazomet-treated plots without the fungus). Amendment with DoUCR50 reduced *H. schachtii* population densities in the first growing year of application compared to their respective Dazomet control in Schach1. This find confirmed the positive suppressive effects against *H. schachtii* of DoUCR50 in fumigated soil under greenhouse and microplot conditions (Olatinwo et al., 2006a, b). In California, *H. oviparasitica* was considered the major agent in suppressing root-knot nematodes in a peach orchard, and its’ close relative DoUCR50 was shown to be one of the most abundant fungi in *H. schachtii* cysts from the suppressive 9E soil (Stirling et al., 1979; Westphal and Becker, 2001; Yin et al., 2003). In the microplots discussed here, this difference was not maintained in the subsequent cropping cycles, suggesting that the suppressiveness due to DoUCR50 did not become continually established in this context. There was no indication for long-term establishment of this fungus within the nematode populations because no additional differences in egg health or population densities compared to other treatments were detected. In its geographical origin, a single application of the fungus led to a stable *H. schachtii* suppressiveness over the entire experimental period (Olatinwo et al., 2006a). That success of DoUCR50 in parasitizing nematodes was associated with its ability to occupy the rhizoplane of host plants (Olatinwo et al., 2006a). Recolonization of the soil by microorganisms after the biocidal treatment may have interfered with the persistence of DoUCR50 although some suppressive capacity of the fungus was found when it was co-inoculated with the nematode (Olatinwo et al., 2006b). Even in its original ecosystem in California, low levels of DoUCR50 in *H. schachtii* cysts from field suppressive soils were found by Yang et al. (2012), suggesting that other microorganisms competed with DoUCR50 in the cysts and eventually replaced this fungus. Also, this fungus was exposed to overwintering conditions of freezing soil temperatures in Germany that it presumably never experiences in its original California niche. The incapacity for microbial establishment after amendment with inocula is not new. For example, populations of rhizobacteria introduced on seed or into the soil to persist in time after a successful establishment, and their eventual decrease, illustrates the great impact of biological buffering (Kluepfel, 1993; Weller and Thomashow, 1994).

The obligate hyperparasite *P. nishizawae* did not establish in these experiments, and had limited measurable initial effects following application. A slight yield improvement over time was only observed for Schach1 in the susceptible cultivar. This bacterium had first been isolated from *Heterodera glycines*, a close relative of *H. schachtii*, but the sensitivity of the nematode and the capacity of the bacterium to complete its lifecycle was not comprehensively studied (Noel et al., 2005). Thus, its efficacy may have been encumbered by its lack of infectivity on *H. schachtii*. The release of the bacterium into a quite different environment than its original niche in the Midwest of the United States may also have influenced its lack of establishment capacity. *P. nishizawae* is endoparasitic, and thus it does not grow outside of the nematode but its spores are subjected to myriads of soil organisms. Chen and Dickson (1998) have suggested the possibility that microfauna feed on spores of *Pasteuria penetrans* in field soil, especially at high spore density. Talavera et al. (2002) suggested that amoebae and rotifers could have fed on spores of the close relative *P. penetrans*. Although watering can have a positive distribution effect on spores of *P. penetrans* (Talavera et al., 2002), their downward dispersal with percolating water resulting from rainwater or irrigation can lead to a depletion of spores in the top 15–20 cm of soil if they are not continuously amplified in that soil layer (Cetintas and Dickson, 2005). Leaching of endospores is also greater in sandy than in clay soils. Under a drip system, 76% of endospores leached 10 cm after 24 h in sand. With increasing clay content fewer endospores leached, since spores got trapped within clay aggregates (Dabire and Mateille, 2004). We did not trace the re-distribution of spores of *P. nishizawae* but 4.4% of clay content at Münster was below the percentages considered to be optimal for biological control with *P. penetrans* (10–30%).

The generally expected parasite–host plant interaction remained in place that initial host plant root penetration was related to number of nematode eggs in the soil. The effects of these numbers on early plant growth were less clearly related than reported for the same sugar beet genotypes when healthy nematode populations were used (Westphal, 2013). In Westphal’s studies, early juvenile penetration of sugar beet roots predicted the early canopy expansion. Canopy diameter was also predictive of final yield for the susceptible Beretta. Here, this lack of association of nematode population densities and early plant measure, and then final yield further illustrate the reduction of nematode infectivity and damage potential throughout the monitored growing seasons.

## Conclusion

A suppressive effect of the monoculture was evident by the rapid and dramatic increase in diseased eggs that constituted over 90% of the total eggs by the third cropping cycle in both pathotypes. Irrespective of the cultivar that was cropped, this pronounced increase in diseased eggs suggests the compatibility of controlling factors with different sugar beet genotypes. Amendments with DoUCR50 had only transient effects on *H. schachtii* population densities or yield while the naturally developing suppressiveness appeared stable and more effective in supporting yields. The obligate bacterium *P. nishizawae* failed to be active under the conditions described here. The use of resistant and especially tolerant cultivars supported the development of suppressiveness, and the use of tolerant cultivars could overcome the yield penalty of generating soil suppressiveness by monoculture of susceptible hosts.

## Data Availability Statement

The datasets generated for this study are available on request to the corresponding author.

## Author Contributions

CE obtained partial funding, designed the experiments, conducted the research, wrote the draft, edited the manuscript, and conducted analysis. HH edited the manuscript. AW designed the project, conducted the research, mentored the first author, conducted part of the analysis, edited the manuscript, and obtained the research effects.

## Conflict of Interest

The authors declare that the research was conducted in the absence of any commercial or financial relationships that could be construed as a potential conflict of interest.

## Supplementary Material

FIGURE S1 Monthly and vegetation period (April to October) averages and sums **(a)** air temperature, and **(b)** precipitation in Münster, Germany during 2010–2013. Data obtained from WetterKontor GmbH, online: wetterkontor.de; accessed: May 28, 2020.

FIGURE S2 Final population densities of **(a)** healthy eggs, **(b)** diseased eggs, and **(c)** total eggs of *Heterodera schachtii* Schach0 in Münster from 2010 to 2013 under sugar beet cropping (Beretta, Sanetta, Pauletta), and averaged across treatment and soil depth. Data are presented as back-transformed lsmeans ± lsmse. Bars within each year indexed with the same letter were not significantly different at *P* = 0.05; a–c: 2010; d–f: 2011; g–i: 2012; j–l: 2013. Bars within each cultivar over year indexed with the same letter were not significantly different at *P* = 0.05; A–D: Beretta; E–H: Sanetta; I–L: Pauletta.

FIGURE S3 Final population densities of **(a)** healthy eggs, **(b)** diseased eggs, and **(c)** total eggs of *Heterodera schachtii* Schach1 in Münster, from 2010 to 2013 under sugar beet cropping (Beretta, Sanetta, Pauletta), and averaged across treatment and soil depth. Data are presented as backtransformed lsmeans ± lsmse. Bars within each year indexed with the same letter were not significantly different at *P* = 0.05; a–c: 2010; d–f: 2011; g–i: 2012; j–l: 2013. Bars within each cultivar over year indexed with the same letter were not significantly different at *P* = 0.05; A–D: Beretta; E–H: Sanetta; I–L: Pauletta.

FIGURE S4 Final population densities of **(a)** healthy eggs, **(b)** diseased eggs, and **(c)** total eggs of *Heterodera schachtii* Schach1 in Münster, from 2010 to 2013 under four different treatments averaged across cultivar and soil depth. Data are presented as backtransformed lsmeans ± lsmse. Bars within each year indexed with the same letter were not significantly different at *P* = 0.05; a–d: 2010; e–h: 2011; i–l: 2012; m–p: 2013. Bars within each treatment over year indexed with the same letter were not significantly different at *P* = 0.05; A–D: untreated control (Control); E–H: *P. nishizawae* (*P. nish*); I–L: Dazomet + *Hyalorbilia* sp. strain DoUCR50 (Daz + DoUCR50); M–P: Dazomet – *Hyalorbilia* sp. strain DoUCR50 (Daz – DoUCR50).

TABLE S1 Lsmeans ± lsmse of early plant growth and early root invasion by nematodes in an experiment with sugar beet and *Heterodera schachtii* Schach0 and Schach1 at Münster, Germany, from 2010 to 2013.
