# Supplementary figures and images for: Biological Suppression of Populations of Heterodera schachtii Adapted to Different Host Genotypes of Sugar Beet
Source: Front Plant Sci. 2020 Jun 19;11:812. doi: 10.3389/fpls.2020.00812 (PMC7317003; doi:10.3389/fpls.2020.00812)

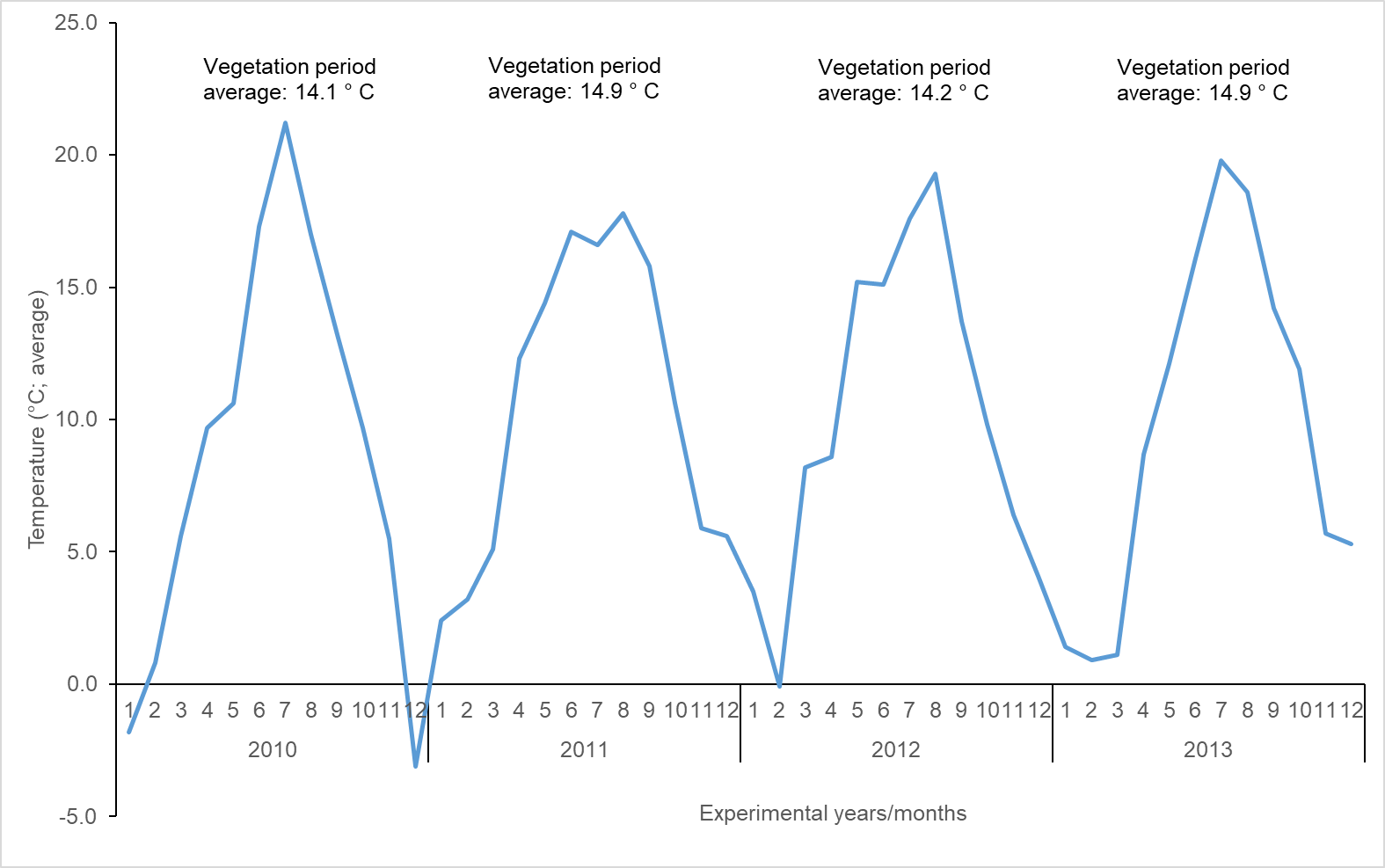

Supplement: FIGURE S1 — Monthly and vegetation period (April to October) averages and sums (a) air temperature, and (b) precipitation in Münster, Germany during 2010–2013. Data obtained from WetterKontor GmbH, online: wetterkontor.de; accessed: May 28, 2020. [file Data_Sheet_1.zip › SUP/Figure S1a.TIFF]

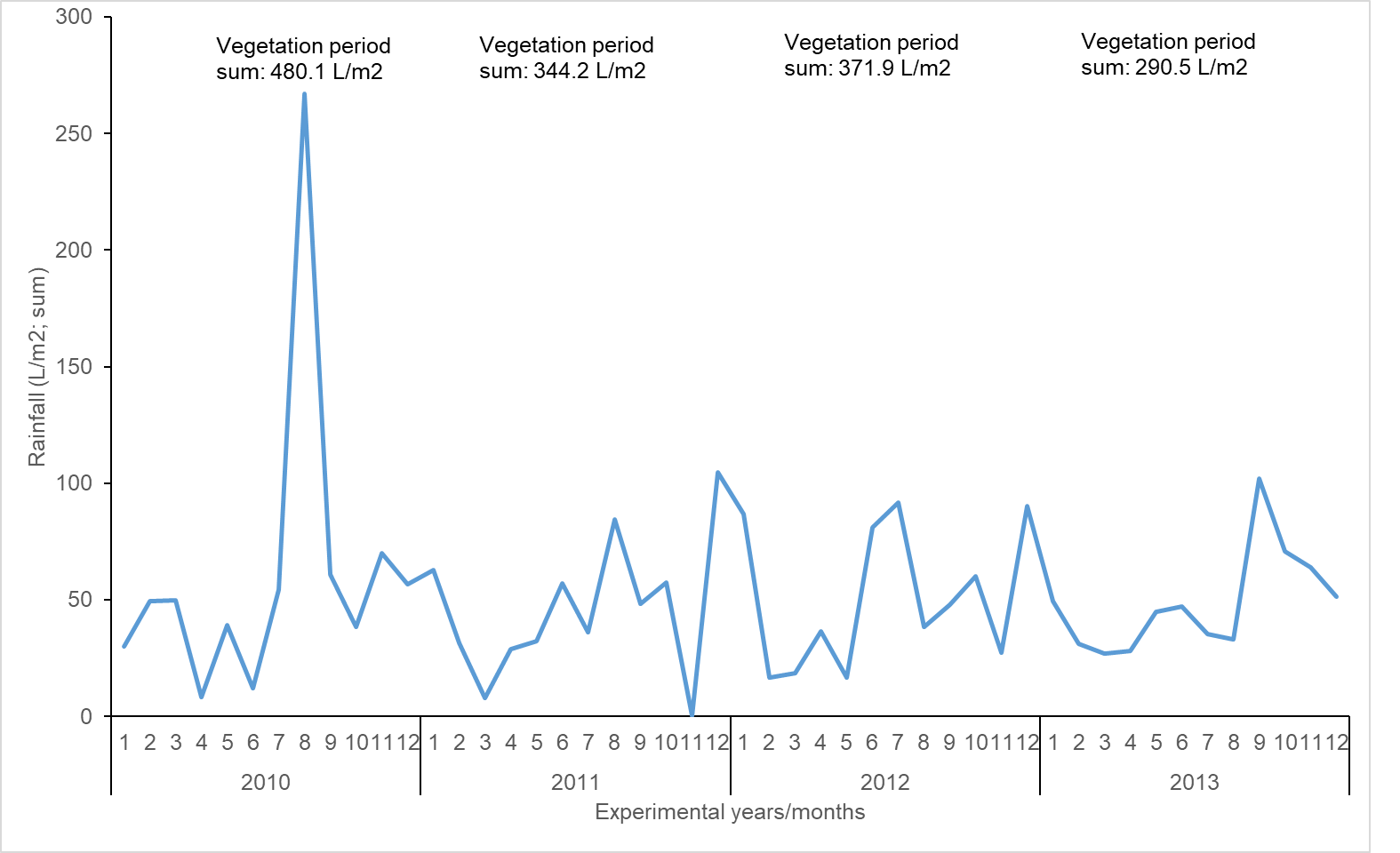

Supplement: FIGURE S1 — Monthly and vegetation period (April to October) averages and sums (a) air temperature, and (b) precipitation in Münster, Germany during 2010–2013. Data obtained from WetterKontor GmbH, online: wetterkontor.de; accessed: May 28, 2020. [file Data_Sheet_1.zip › SUP/Figure S1b.TIFF]

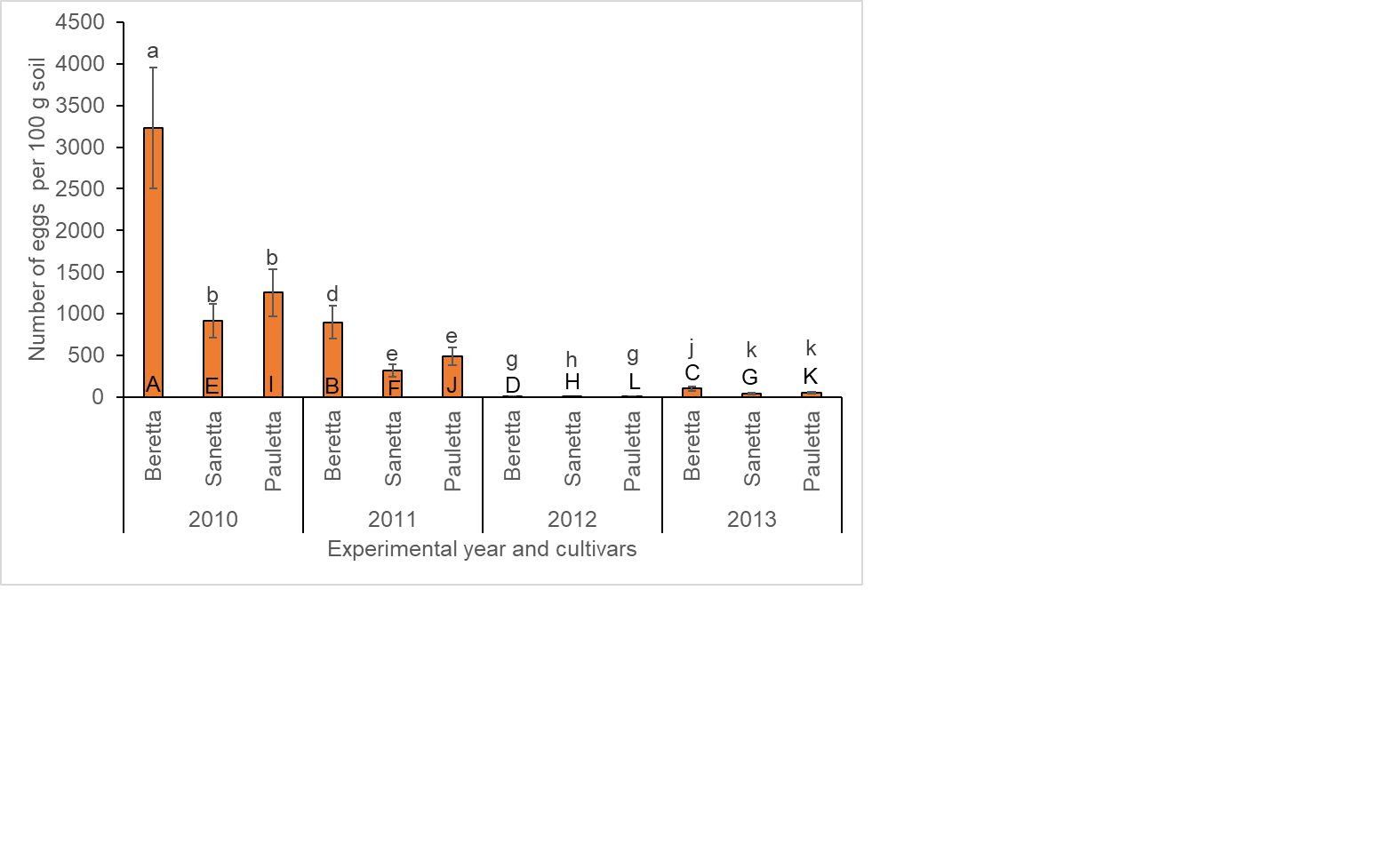

Supplement: FIGURE S1 — Monthly and vegetation period (April to October) averages and sums (a) air temperature, and (b) precipitation in Münster, Germany during 2010–2013. Data obtained from WetterKontor GmbH, online: wetterkontor.de; accessed: May 28, 2020. [file Data_Sheet_1.zip › SUP/Figure S2a.TIFF]

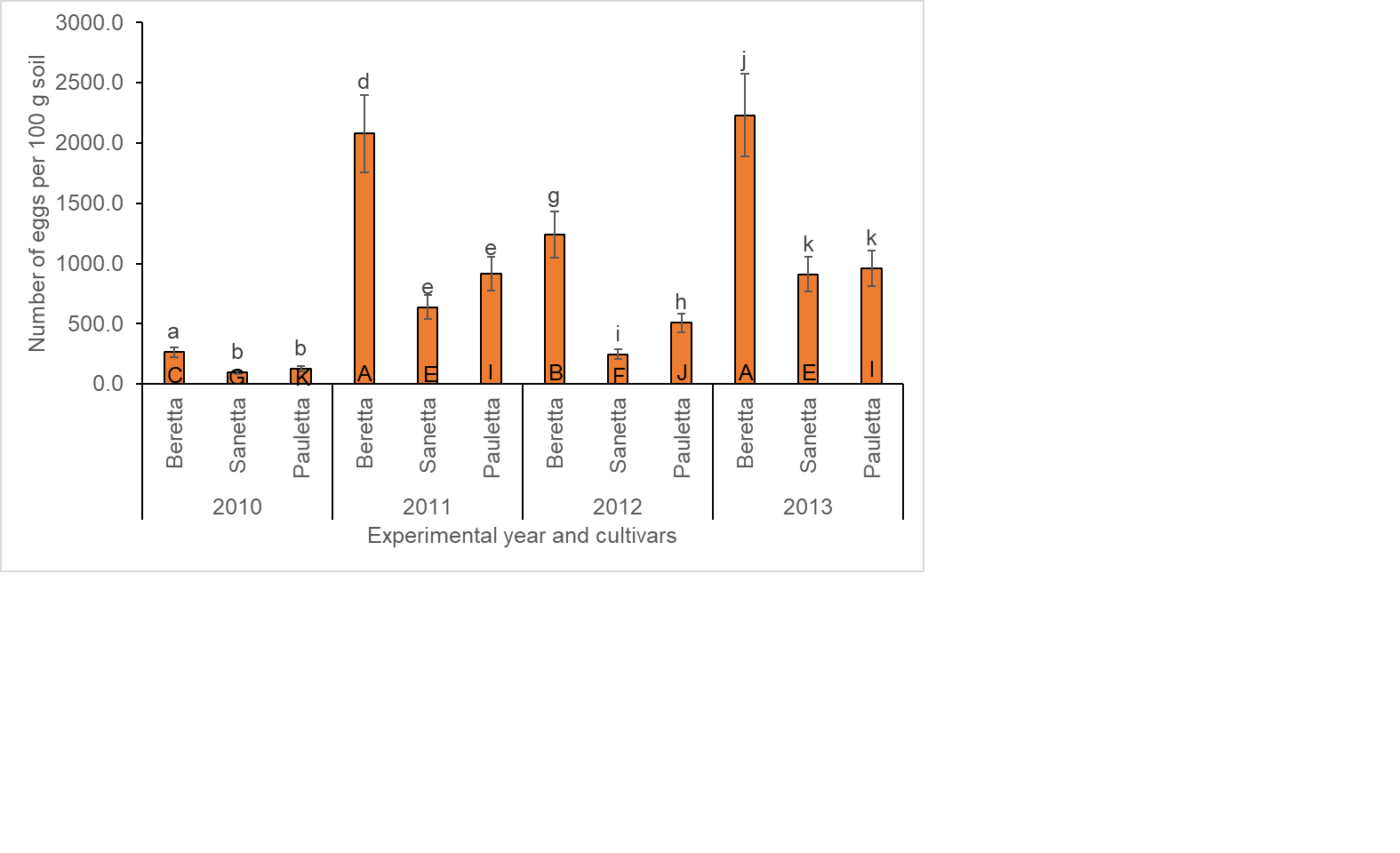

Supplement: FIGURE S1 — Monthly and vegetation period (April to October) averages and sums (a) air temperature, and (b) precipitation in Münster, Germany during 2010–2013. Data obtained from WetterKontor GmbH, online: wetterkontor.de; accessed: May 28, 2020. [file Data_Sheet_1.zip › SUP/Figure S2b.TIFF]

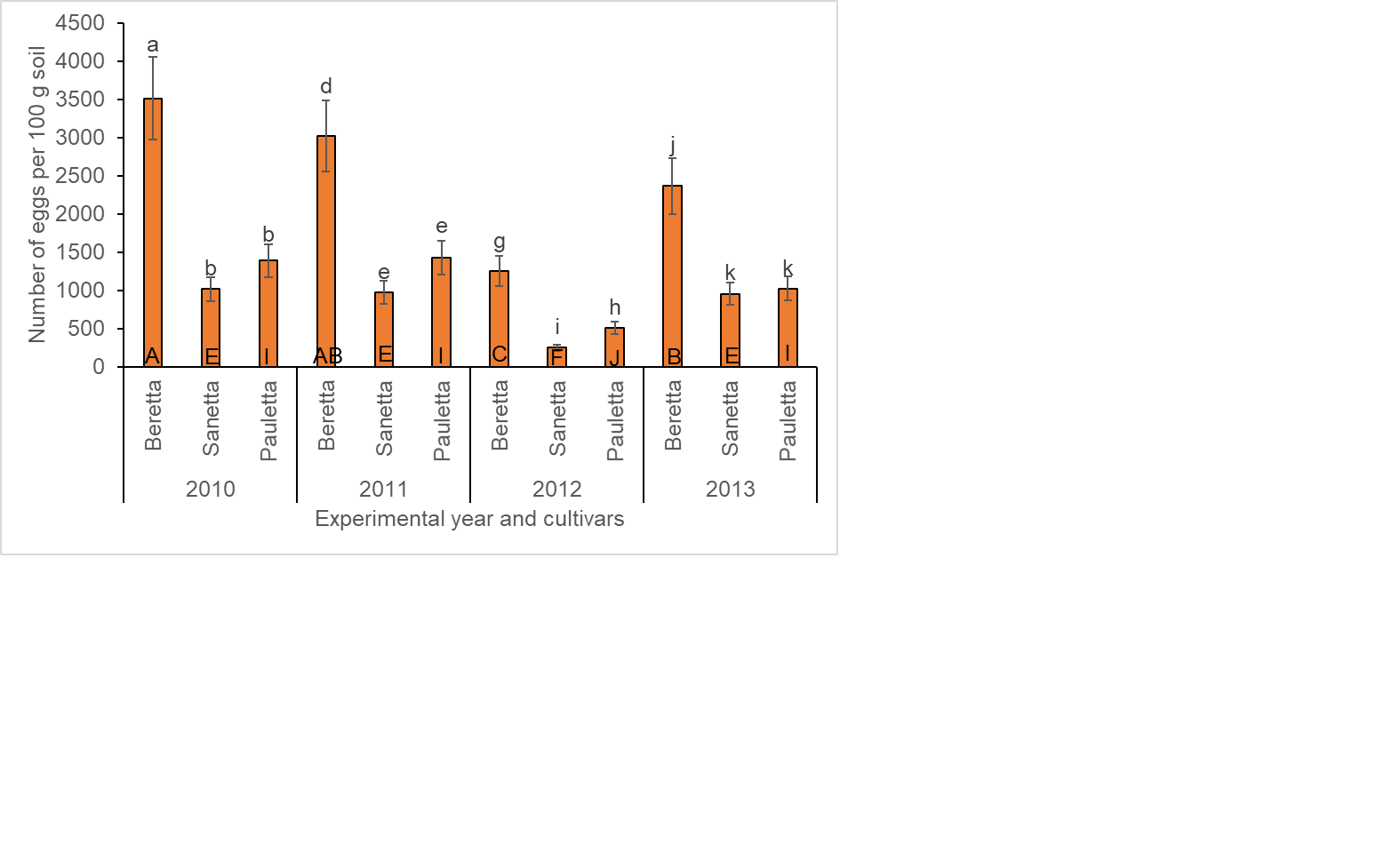

Supplement: FIGURE S1 — Monthly and vegetation period (April to October) averages and sums (a) air temperature, and (b) precipitation in Münster, Germany during 2010–2013. Data obtained from WetterKontor GmbH, online: wetterkontor.de; accessed: May 28, 2020. [file Data_Sheet_1.zip › SUP/Figure S2c.TIFF]

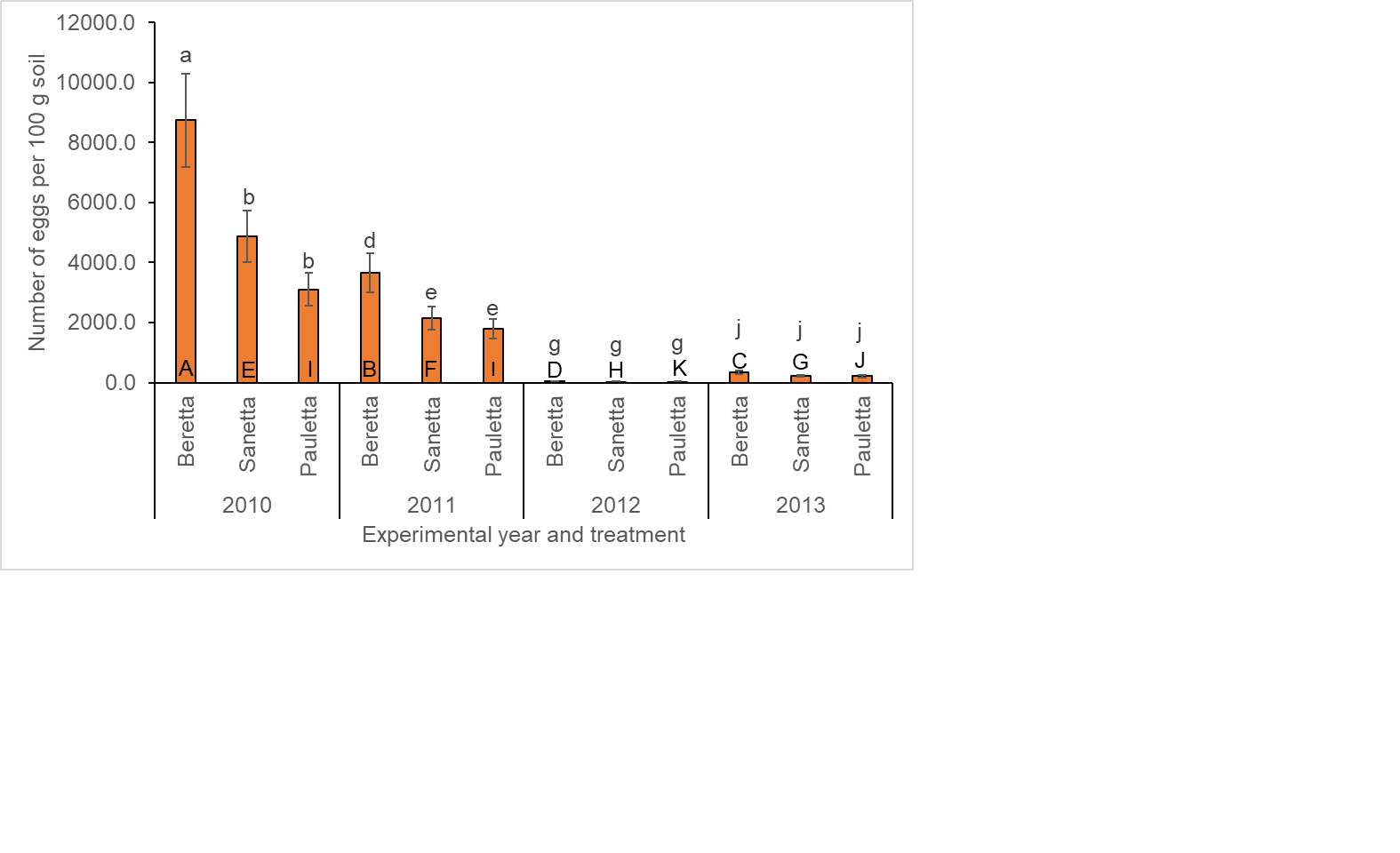

Supplement: FIGURE S1 — Monthly and vegetation period (April to October) averages and sums (a) air temperature, and (b) precipitation in Münster, Germany during 2010–2013. Data obtained from WetterKontor GmbH, online: wetterkontor.de; accessed: May 28, 2020. [file Data_Sheet_1.zip › SUP/Figure S3a.TIFF]

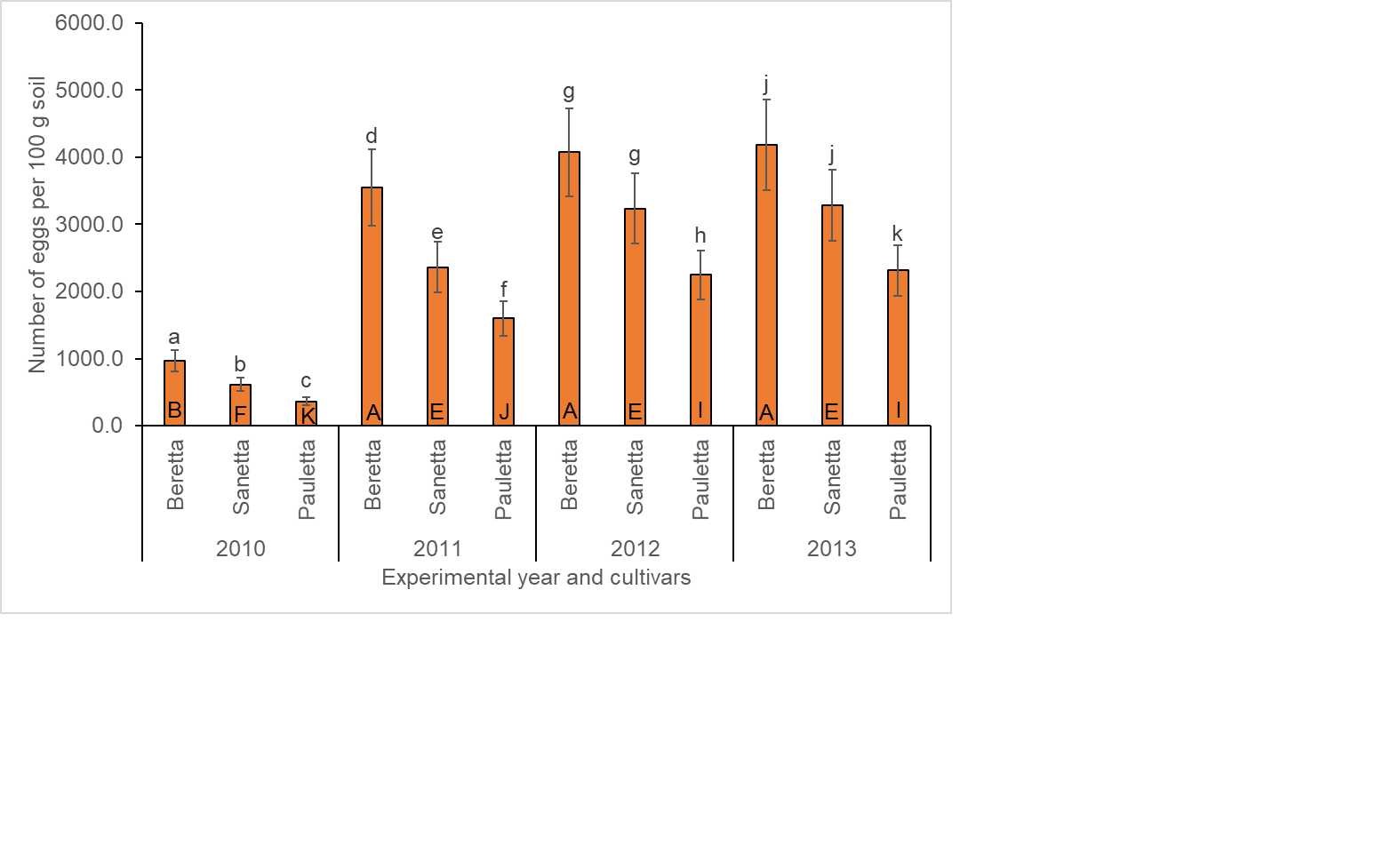

Supplement: FIGURE S1 — Monthly and vegetation period (April to October) averages and sums (a) air temperature, and (b) precipitation in Münster, Germany during 2010–2013. Data obtained from WetterKontor GmbH, online: wetterkontor.de; accessed: May 28, 2020. [file Data_Sheet_1.zip › SUP/Figure S3b.TIFF]

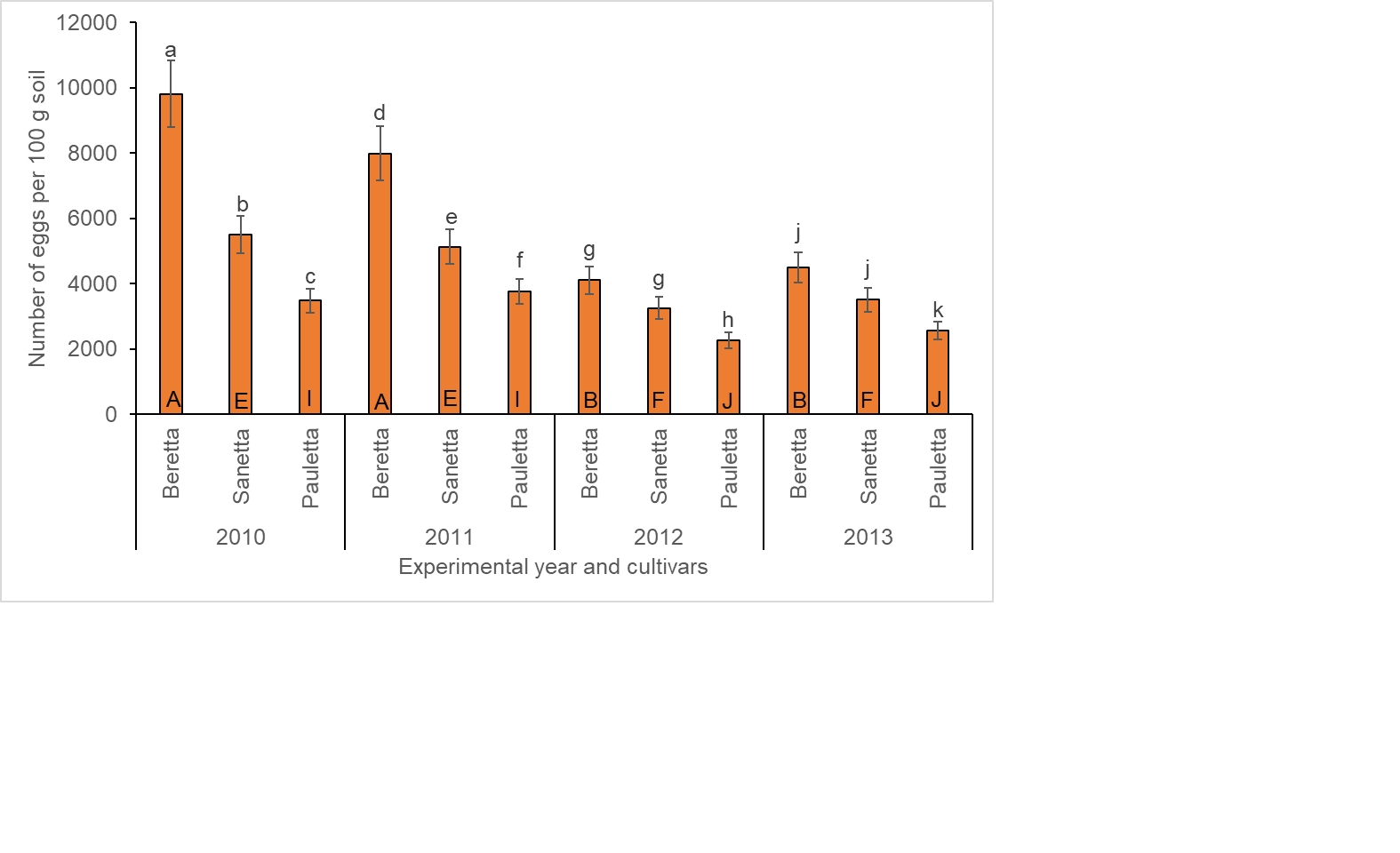

Supplement: FIGURE S1 — Monthly and vegetation period (April to October) averages and sums (a) air temperature, and (b) precipitation in Münster, Germany during 2010–2013. Data obtained from WetterKontor GmbH, online: wetterkontor.de; accessed: May 28, 2020. [file Data_Sheet_1.zip › SUP/Figure S3c.TIFF]

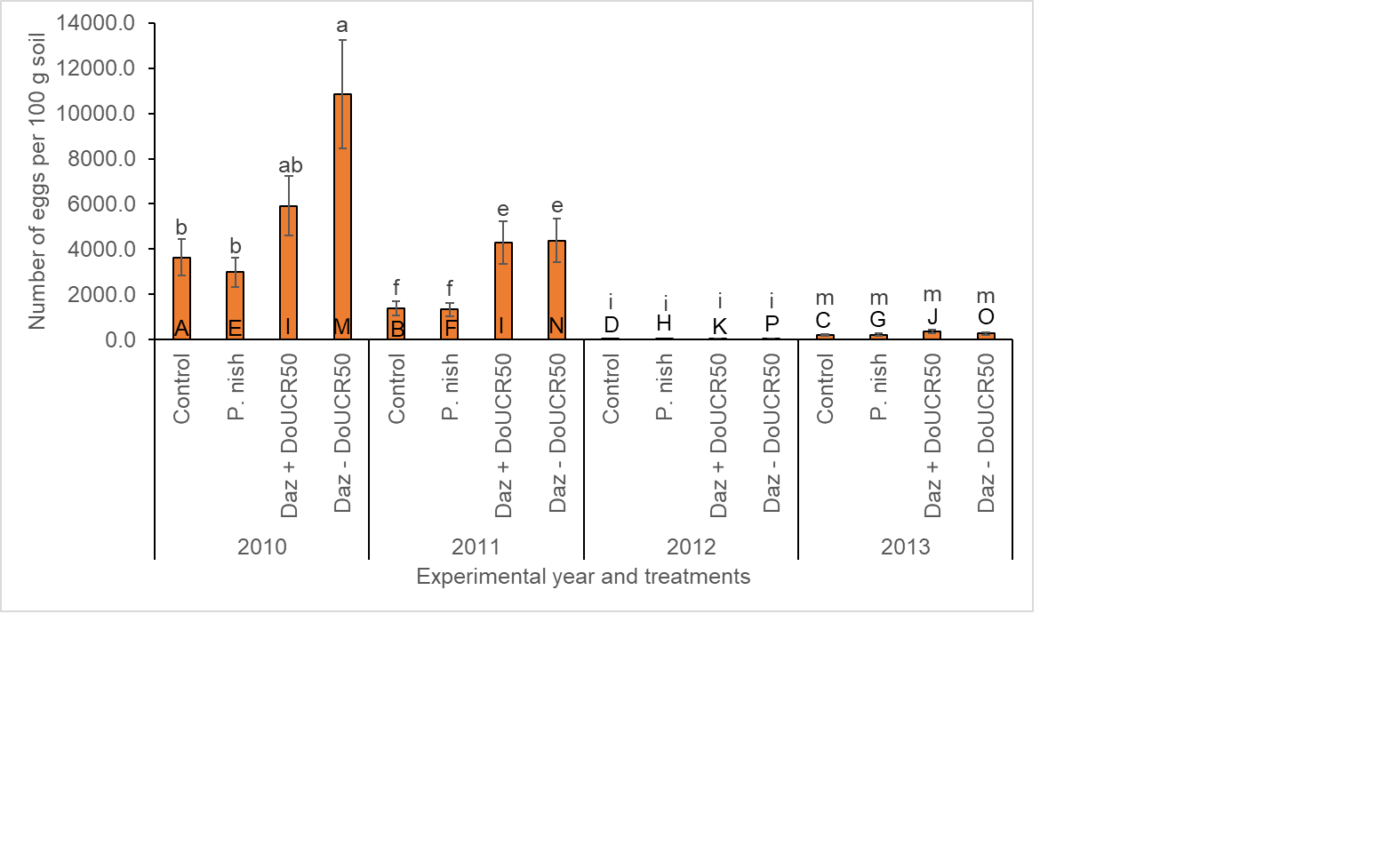

Supplement: FIGURE S1 — Monthly and vegetation period (April to October) averages and sums (a) air temperature, and (b) precipitation in Münster, Germany during 2010–2013. Data obtained from WetterKontor GmbH, online: wetterkontor.de; accessed: May 28, 2020. [file Data_Sheet_1.zip › SUP/Figure S4a.TIFF]

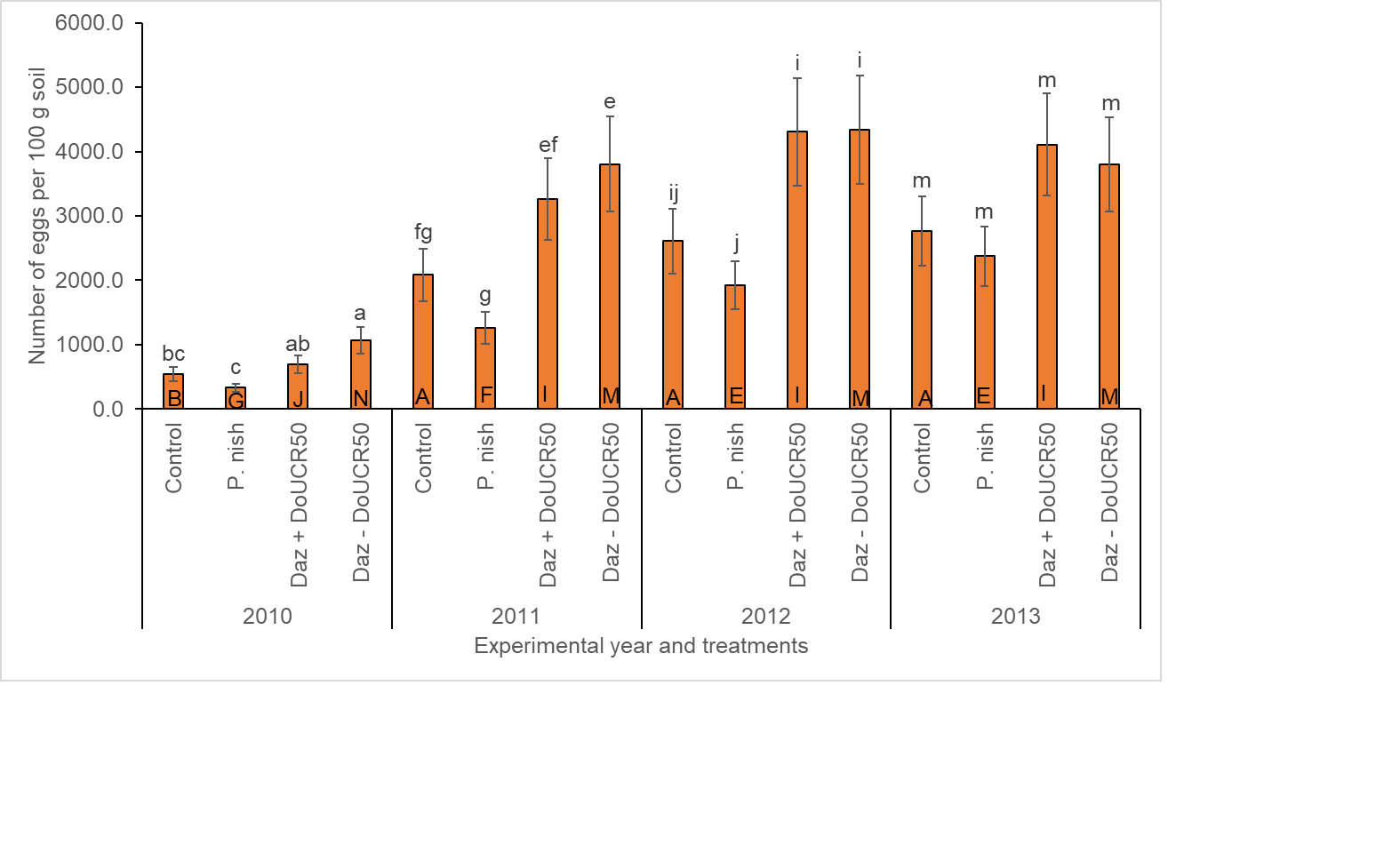

Supplement: FIGURE S1 — Monthly and vegetation period (April to October) averages and sums (a) air temperature, and (b) precipitation in Münster, Germany during 2010–2013. Data obtained from WetterKontor GmbH, online: wetterkontor.de; accessed: May 28, 2020. [file Data_Sheet_1.zip › SUP/Figure S4b.TIFF]

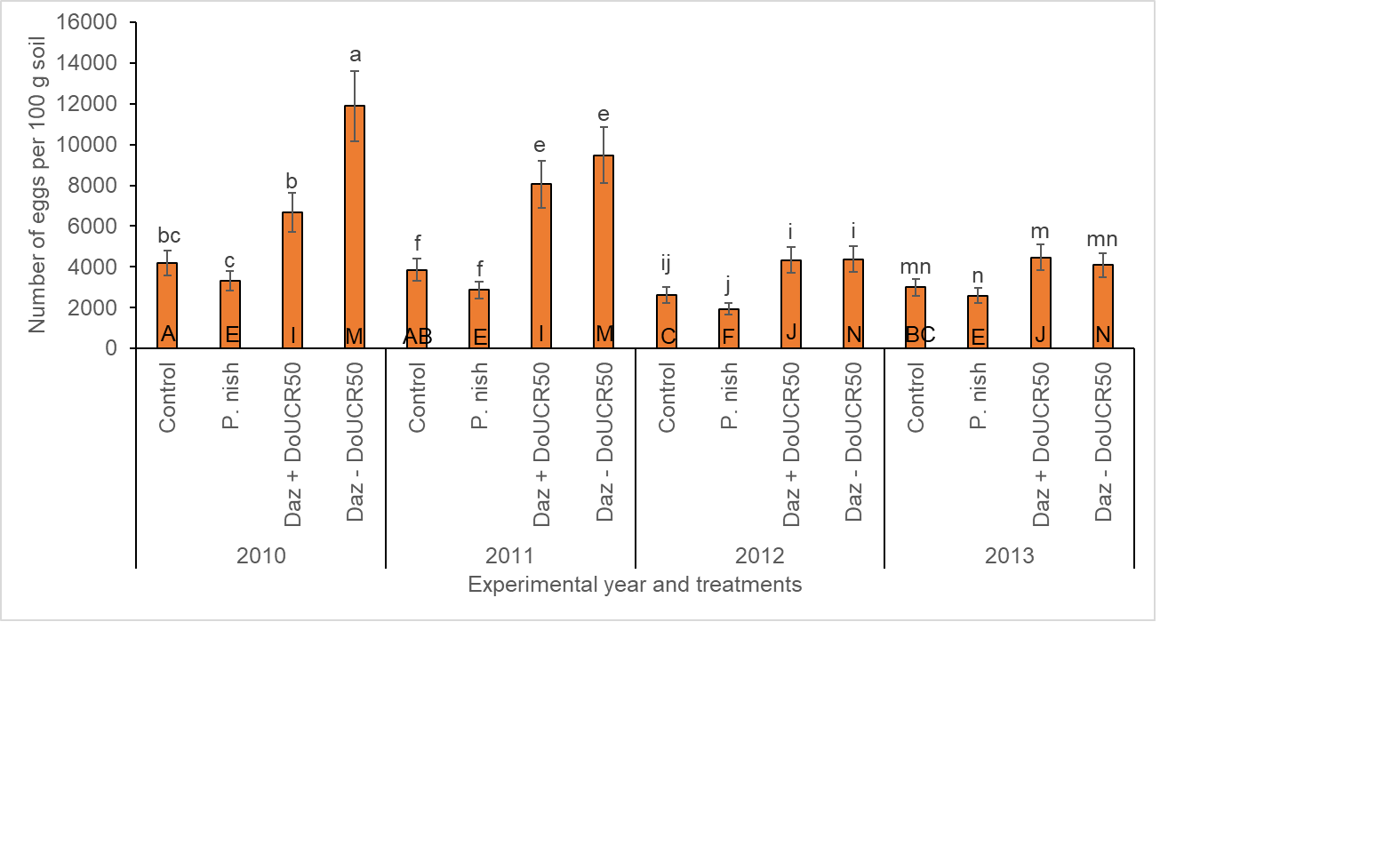

Supplement: FIGURE S1 — Monthly and vegetation period (April to October) averages and sums (a) air temperature, and (b) precipitation in Münster, Germany during 2010–2013. Data obtained from WetterKontor GmbH, online: wetterkontor.de; accessed: May 28, 2020. [file Data_Sheet_1.zip › SUP/Figure S4c.TIFF]
