# Supplementary material for: Biological Suppression of Populations of Heterodera schachtii Adapted to Different Host Genotypes of Sugar Beet
Source: Front Plant Sci. 2020 Jun 19;11:812. doi: 10.3389/fpls.2020.00812 (PMC7317003; doi:10.3389/fpls.2020.00812)
Supplement: TABLE S1 — Lsmeans ± lsmse of early plant growth and early root invasion by nematodes in an experiment with sugar beet and Heterodera schachtii Schach0 and Schach1 at Münster, Germany, from 2010 to 2013. [file Table_1.DOC]

Table S1 Lsmeans ± lsmse of early plant growth and early root invasion by nematodes in an experiment with sugar beet and *Heterodera schachtii* Schach0 and Schach1at Münster, Germany from 2010 to 2013.

|  |  |  |  | | |  |  | | |  |
| --- | --- | --- | --- | --- | --- | --- | --- | --- | --- | --- |
|  |  |  | Canopy diameter | | |  | Early root invasion by nematodes | | |  |
|  |  |  | (cm) | | |  | (J2 per cm of root) | | |  |
|  |  |  |  |  |  |  |  |  |  |  |
| Pathotype | Year |  | Beretta | Sanetta | Pauletta |  | Beretta | Sanetta | Pauletta |  |
|  |  |  |  | | |  |  | | |  |
| Schach0 | 2010 |  | 46.9 ± 1.4 | 41.4 ± 1.2 | 52.1 ± 1.5 |  | 1.9 ± 0.3 | 1.0 ± 0.2 | 0.9 ± 0.2 |  |
|  | 2011 |  | 25.7 ± 0.8 | 25.1 ± 0.8 | 30.8 ± 0.9 |  | 8.9 ± 1.2 | 3.6 ± 0.5 | 3.1 ± 0.5 |  |
|  | 2012 |  | 31.5 ± 0.9 | 31.3 ± 0.9 | 36.2 ± 1.1 |  | 4.6 ± 0.7 | 2.1 ± 0.4 | 1.7 ± 0.3 |  |
|  | 2013 |  | 44.2 ± 1.3 | 38.8 ± 1.1 | 48.3 ± 1.4 |  | 10.6 ± 1.4 | 5.7 ± 0.8 | 6.0 ± 0.8 |  |
| Schach1 | 2010 |  | 59.9 ± 1.6 | 58.6 ± 1.5 | 68.5 ± 1.8 |  | 1.0 ± 0.3 | 0.7 ± 0.2 | 0.4 ± 0.2 |  |
|  | 2011 |  | 22.2 ± 0.6 | 21.0 ± 0.6 | 26.5 ± 0.7 |  | 11.2 ± 1.8 | 7.3 ± 1.2 | 5.1 ± 0.9 |  |
|  | 2012 |  | 32.1 ± 0.9 | 30.1 ± 0.8 | 37.2 ± 1.0 |  | 7.1 ± 1.2 | 7.6 ± 1.2 | 5.5 ± 0.9 |  |
|  | 2013 |  | 43.5 ± 1.1 | 39.6 ± 1.0 | 48.3 ± 1.3 |  | 7.8 ± 1.3 | 6.3 ± 1.0 | 3.9 ± 0.7 |  |
